# Validation of the Measurement of Need Frustration

**DOI:** 10.3389/fpsyg.2019.01742

**Published:** 2019-07-30

**Authors:** Isabeau K. Tindall, Guy J. Curtis

**Affiliations:** ^1^Discipline of Psychology, Murdoch University, Murdoch, WA, Australia; ^2^Discipline of Psychology, Murdoch University, and School of Psychological Science, The University of Western Australia, Perth, WA, Australia

**Keywords:** need satisfaction, need frustration, NSFS, ill-being, anxiety, depression, stress, well-being

## Abstract

Until recently, need frustration was considered to be the absence of need satisfaction, rather than a separate dimension. Whilst the absence of need satisfaction can hamper growth, experiencing need frustration can lead to malfunctioning and subsequent psychopathology. Therefore, examining these constructs separately is vital, as they produce different outcomes, with the consequences of need frustration potentially more severe. This study sought to examine predictors of need frustration using undergraduate students and individuals from the wider community (*N* = 510, females *N =* 404, *M*_age_ = 24.15). Participants completed the new need satisfaction frustration scale and measures of anxiety, stress, depression, and negative and positive affect. Support for the position that need frustration is separate to Need Satisfaction and is related to psychological health problems (i.e., ill-being) was found. However, autonomy frustration was not found to be a significant predictor of ill-being. Extending previous research, this study found relationships of stress and somatic anxiety with need frustration. Further, a relationship between need frustration with anxiety and depression occurred, when these symptom dimensions were examined separately, through distinct questionnaires. Support for the construct of need frustration highlights the necessity of examining need frustration in addition to need satisfaction within future studies. Interventions specific to reducing need frustration, specifically competence and relatedness frustration within both the educational and workplace setting are outlined.

## Introduction

According to basic needs theory (BNT), a mini-theory of Self-Determination Theory (Deci and Ryan, 2000), individuals are motivated by three key psychological needs, the need for: autonomy, competence and relatedness. Autonomy is defined as the perception of control over one’s behavior rather than feeling controlled by external factors. Competence relates to an individual’s belief in their ability to attain desired outcomes, and Relatedness, the degree to which an individual feels closeness and a sense of belonging with others. BNT contends that meeting these needs are necessary for optimal human development (Ryan et al., 1996). The degree to which these needs are met, has direct implications to both the educational and workplace setting (education: Copeland and Levesque-Bristol, 2011; workplace: Gagné et al., 1997; Baard et al., 2004). Need satisfaction over these three domains, are strong predictors of first year retention rates, the perception of the university setting as a positive learning environment and increased academic performance (Copeland and Levesque-Bristol, 2011). Whilst, within the workplace, increased need satisfaction is related to better job performance, improved psychological well-being (Baard et al., 2004), increased work motivation (Gagné et al., 1997), and stronger employee commitment (Gagné et al., 2008). According to BNT the degree to which needs are met over the domains of autonomy, competency and relatedness, directly relate to a sense of subjective wellbeing, whilst having these needs frustrated, leads to ill-being. Therefore, according to this theory, need frustration is not a separate construct in and of itself, but rather, occurs because of the absence of need satisfaction (Deci and Ryan, 2000).

More recent research, however, has speculated that need frustration is not just the inverse of need satisfaction, but rather, is a distinct construct (Deci and Ryan, 2000; Sheldon and Gunz, 2009; Bartholomew et al., 2011a; Longo et al., 2016). Supporting this assertion, Longo et al. (2016) stated that these two constructs have separate theoretical underpinnings, and therefore, will predict different outcomes. Specifically, satisfaction related to the domains of autonomy, competence and relatedness, is associated with positive outcomes (well-being) such as positive affect. Whilst frustration related to these three domains (need frustration) can predict negative outcomes (ill-being) such as negative affect, depression and anxiety (Bartholomew et al., 2011a). Their study found support for this theory by showing significant relationships existed between satisfaction and positive outcomes, and frustration and negative outcomes. The study by Longo et al. (2016) is therefore, an extension of BNT (Deci and Ryan, 2000) due to the further refinement as to what defines, and predicts, satisfaction and frustration. Specifically, it was found that ill-being was uniquely predicted by experiences of need frustration and not from merely experiencing low need satisfaction (Longo et al., 2016). A recent study by Longo et al. (2018) found further support for the perspective that need satisfaction and need frustration are distinct constructs.

Due to the recency of the proposition that need frustration is separate from need satisfaction, limited research has been conducted into need frustration, and therefore, correlates of this construct. Research into need frustration is imperative; although a lack of need satisfaction is related to negative outcomes, increased need frustration is considered especially harmful, and linked to potential psychopathology (Bartholomew et al., 2011a). According to Vansteenkiste and Ryan (2013), this is illustrated through an example juxtaposing the repercussions of low need satisfaction as compared to experiencing increased need frustration within the workplace. An individual experiencing low need satisfaction through reduced relatedness with colleagues, might not feel as excited about their work. However, an individual actively bullied, ridiculed and excluded by colleagues, therefore, also experiencing low relatedness through high degrees of need frustration, will be at the additional risk of developing psychopathology, such as depression and severe stress. It can then be said that although a lack of need satisfaction can lead to a lack of fulfillment, need frustration, is also strongly related to malfunctioning (Vansteenkiste and Ryan, 2013). Given that the degree of mental illness has been steadily increasing within both the workplace (Bonde, 2008; Fan et al., 2015) and educational setting (American College Health Association, 2018), it is important to examine the potential link between need frustration and psychopathology.

In light of the above research, the present study aimed to further examine the relationship between psychological health problems, and need frustration. We did this through examining the relationship between the need satisfaction and frustration scale (NSFS) and ill-being found by Longo et al. (2016) through examining the factor structure of the NSFS. Particular attention was given to the predictors of need frustration due to the scarcity of research into this factor. Need satisfaction was not the focus of the present study as extensive research has been conducted into this construct (Deci and Ryan, 2002; Baard et al., 2004). Therefore, an omnibus of negative emotionality measures expected to be predicted by need frustration were included in this study. Negative emotionality measures of anxiety, stress, depression and negative affect, were included.

A limitation of the Longo et al. (2016) study, was the lack of ability to distinguish between anxiety and depression manifested through increased ill-being. Longo et al. (2016) used the General Health Questionnaire (GHQ, Goldberg and Williams, 1988) to measure the influence of ill-being on anxiety and depression, through the Anxiety-Depression subscale, which does not separate between these constructs. Although anxiety and depression share variance related to general distress, they are theoretically distinct dimensions, with anxiety uniquely related to social tension/arousal, and depression; anhedonia/low affect (Clark and Watson, 1991). Therefore, to allow for an examination of the distinct relationship between anxiety and depression with ill-being, we included well validated measures of both anxiety and depression. To allow for replication of Longo et al. (2016), a measure of negative affect was also included. Although an investigation into need frustration has already occurred within the sporting context (Bartholomew et al., 2011b), and within general life (Sheldon and Gunz, 2009), the present study sought to directly extend on the study by Longo et al. (2016). Therefore, we examined the influence of need frustration on negative emotionality specifically within the context of the educational setting and workplace. According to research into the educational setting (Riolli et al., 2012), university students experience high levels of stress. Further, the workplace can be inherently stressful (Colligan and Higgins, 2006). Therefore, in extension of Longo et al. (2016), in addition to measures of negative affect, depression and anxiety, we also included a measure of stress.

In light of the above aim, it was hypothesized that:

(1) Measures of psychological health problems, specifically those measuring depression, anxiety, stress and negative affect, will negatively relate to satisfaction and positively relate to frustration.
(2) Need Satisfaction will be positively correlated with positive affect.
(3) That measures related to psychological health problems and positive affect will be strongly related to their proposed factors, highlighting support for the factor structure put forward by Longo et al. (2016).

## Materials and Methods

### Participants

A sample of 510 (females *N =* 404, *M*_age_ = 24.15, *SD* = 8.06; range = 18–59), undergraduate students from Murdoch University and members of the wider community (78% Caucasian) participated in this study for partial course credit, or the potential to gain a gift voucher, respectively. Ethics approval was acquired from Murdoch University before data collection.

### Materials

Need satisfaction and frustration scale (NSFS; Longo et al., 2016). The NSFS consists of six 3-item subscales, measuring need satisfaction in the domains of autonomy, relatedness and competence and the other three, measuring need frustration in these domains. This scale examined these needs in the context of work and/or educational settings. Items are rated on a 7-point Likert scale from 1 (*strongly disagree*), to 7 (*strongly agree*), higher scores on the satisfaction subscales indicate greater need satisfaction, whilst higher scores on the frustration subscales indicate increased frustration. An example item is “In my studies/In my job… I feel, I’m given a lot of freedom in deciding how I do things.” The subscales of autonomy, relatedness, and competence over the domains of satisfaction and frustration have exhibited excellent reliability (**α***s* > 0.70) in both the educational and workplace context. Further, the criterion validity of this scale with other measures of need satisfaction are sufficient over both settings (*rs* ≥ 0.4; Longo et al., 2016).

Omnibus affect measures. Anxiety was measured through the State-Trait Inventory for Cognitive and Somatic Anxiety (STICSA; Ree et al., 2008), the State-Trait Anxiety Inventory (STAI; Spielberger et al., 1970, 1983), the Anxiety Sensitivity Index (ASI; Reiss et al., 1986) and the anxiety subscale of the Depression Anxiety Stress Scale-21 (DASS-21; Lovibond and Lovibond, 1995). All anxiety measures exhibited sound validity and internal consistency previously (α*s* > 0.83; Spielberger et al., 1983; Peterson and Heilbronner, 1987; Lovibond and Lovibond, 1995; Grös et al., 2007). Depression was measured through the depression subscale of the DASS-21 (Lovibond and Lovibond, 1995) and the Beck Depression Inventory-II (BDI-II; Beck et al., 1996). For the BDI-II, item 9, “Suicidal Thoughts or Wishes” was removed according to a requirement by the Ethics Committee of Murdoch University. Depression measures included have previously exhibited good validity and internal consistency (α*s* > 0.84; Lovibond and Lovibond, 1995; Dozois et al., 1998). Stress was measured through the stress subscale of the DASS-21 (Lovibond and Lovibond, 1995). This subscale has also exhibited sound internal consistency and reliability (α = 0.90; Lovibond and Lovibond, 1995). Positive affect and negative affect were measured using an adapted version of the Positive and Negative Affect Schedule-X (Watson and Clark, 1994; Church et al., 2014).

Both subscales of positive and negative affect exhibit strong previous internal consistency and reliability (α = 0.83; Watson and Clark, 1994). Response scales for these measures were as they appear in their original sources or manuals.

### Procedure

Participants gave written informed consent and completed questionnaires online. Participants were also told their responses to these questionnaires would be anonymous. The order of questionnaires presented were randomized. Students were recruited through a participant database at Murdoch University and through fliers posted around the university, whilst community members were recruited through social media. The surveys took approximately 30 min to complete.

## Results

Data was non-normal, however, a large sample size was used, and so normality was assumed (Ghasemi and Zahediasl, 2012). Little’s (1988) MCAR test was non-significant and missing values consisted of <5% of the total sample, therefore missing values were imputed with the series mean. Using a *Z* of ±3.29 for assessing outliers (Field, 2009), responses from seven participants were removed. A total of 503 participants were therefore included in the final analysis.

Participants also completed the trait versions of the STICSA cognitive and somatic subscales and the STAI trait, however, these were not included in the analysis of ill-being, as need frustration only theoretically affects state anxiety, as trait anxiety should be stable over time (Spielberger et al., 1983; Ree et al., 2008). Due to overlapping symptom dimensions of the anxiety, depression, stress and negative and positive affect measures, multicollinearity of these measures was checked. No measures exceeded multicollinearity cut offs according to values of the VIF < 10 and tolerance > 0.1 (Hair et al., 1995).

Correlations between the NSFS questionnaire subscales and the measures of interest, as well as descriptive statistics and reliability estimates are reported in Table 1.

**TABLE 1:** Correlations between the six subscales of the NSFS, ill-being, and the PANAS-Positive.

			**Measures**									
			**PANAS positive**	**PANAS negative**	**BDI-II**	**ASI**	**DASS anxiety**	**DASS stress**	**DASS depression**	**STICSA state somatic**	**STICSA state cognitive**	**STAI state**
			
		***M* (SD)**	**32.53 (7.12)**	**22.08 (7.61)**	**13.80 (9.77)**	**22.96 (11.98)**	**9.67 (8.73)**	**14.75 (9.48)**	**10.86 (9.91)**	**15.44 (5.03)**	**19.22 (7.05)**	**41.66 (12.09)**
**Sub-scale**	***M* (SD)**	α	**0.90**	**0.90**	**0.90**	**0.90**	**0.84**	**0.85**	**0.91**	**0.87**	**0.90**	**0.94**
NSFS autonomy satisfaction	14.38 (4.06)	0.87	0.29^∗∗^	–0.27^∗∗^	–0.25^∗∗^	–0.20^∗∗^	–0.19^∗∗^	–0.20^∗∗^	–0.21^∗∗^	–0.22^∗∗^	–0.21^∗∗^	–0.31^∗∗^
NSFS autonomy frustration	11.60 (3.75)	0.75	–0.24^∗∗^	0.27^∗∗^	0.26^∗∗^	0.25^∗∗^	0.18^∗∗^	0.26^∗∗^	0.22^∗∗^	0.15^∗∗^	0.24^∗∗^	0.27^∗∗^
NSFS relatedness satisfaction	13.36 (3.78)	0.80	0.44^∗∗^	–0.39^∗∗^	–0.39^∗∗^	–0.21^∗∗^	–0.25^∗∗^	–0.27^∗∗^	–0.40^∗∗^	–0.18^∗∗^	–0.28^∗∗^	–0.32^∗∗^
NSFS relatedness frustration	12.03 (3.92)	0.76	–0.44^∗∗^	0.51^∗∗^	0.47^∗∗^	0.37^∗∗^	0.38^∗∗^	0.41^∗∗^	0.45^∗∗^	0.26^∗∗^	0.43^∗∗^	0.42^∗∗^
NSFS competence satisfaction	13.81 (3.54)	0.86	0.53^∗∗^	–0.43^∗∗^	–0.44^∗∗^	–0.26^∗∗^	–0.30^∗∗^	–0.30^∗∗^	–0.48^∗∗^	–0.26^∗∗^	–0.42^∗∗^	–0.45^∗∗^
NSFS competence frustration	12.44 (4.01)	0.80	–0.46^∗∗^	0.45^∗∗^	0.46^∗∗^	0.35^∗∗^	0.32^∗∗^	0.40^∗∗^	0.48^∗∗^	0.24^∗∗^	0.44^∗∗^	0.44^∗∗^

^*^p < 0.05, ^∗∗^*p* < 0.01.

As seen in Table 1, internal consistencies for all measures were good, with all alphas >0.7 (Cronbach, 1951). All survey items measuring ill-being outcomes were positively correlated with frustration scales on the NSFS. The PANAS-Positive was positively correlated with satisfaction scales.

**FIGURE 1:**
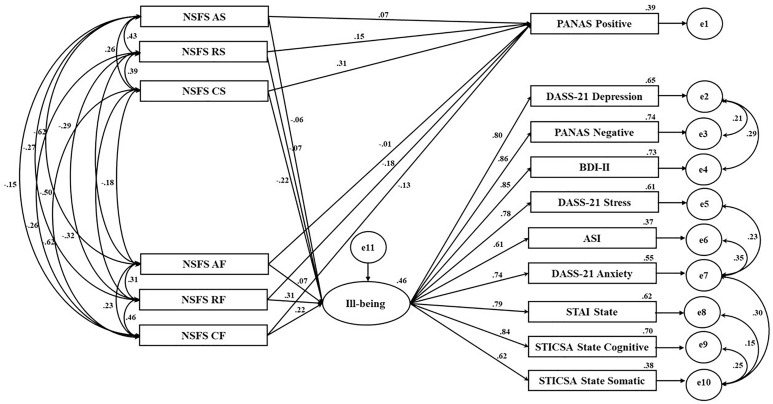
Predicting ill-being outcomes and relationship between satisfaction (NSFS) and positive affect (PANAS-Positive). *A*, autonomy; *S*, satisfaction; *R*, relatedness; *C*, competence; *F*, frustration; *e*, error.

A structural equation model (SEM) was then calculated in AMOS 24 using a maximum likelihood estimation procedure, to assess the factor structure of the NSFS and alignment with the measures of interest (see Figure 1).

Results of the non-constrained model suggested an unacceptable fit: χ^2^(84) = 534.215, *p* < 0.001. CFI = 0. 908, TLI = 0.869, GFI = 0.880, RMR = 3.479, RMSEA = 0.103 (90% CI = 0.095−0.112) and CMIN/DF = 6.360. However, modification indices suggested freeing error variance between the error terms of some of the anxiety, stress, negative affect, and depression measures loading onto ill-being. With regard to correlating the error terms, only measures with strong theoretical support for association were correlated (Cole et al., 2007; Hooper et al., 2008). Subsequently, only errors from measures of anxiety and stress were allowed to correlate (Lovibond and Lovibond, 1995; Roberts et al., 2016), whilst errors from negative affect was only allowed to correlate with depression (Danhauer et al., 2013). For the constrained model, the chi-square value for the overall model fit was significant, χ^2^(77) = 275.996, *p* < 0.001, suggesting a lack of fit between the hypothesized model and the data. However, due to the oversensitivity of the χ^2^ to large sample sizes, other fit indices were assessed (Kline, 1998). Examination of these other indices showed acceptable model fit (Hu and Bentler, 1999; Longley et al., 2005) with CFI = 0.959, TLI = 0.937, GFI = 0.941, RMR = 2.675, CMIN/DF = 3.584 and RMSEA = 0.072 (90% CI = 0.063−0.081).

For frustration items, competence and relatedness frustration loaded significantly onto ill-being. Further, satisfaction items related to competence and relatedness satisfaction loaded significantly onto the PANAS-positive. Autonomy frustration did not load significantly onto ill-being, nor did autonomy satisfaction significantly load onto positive affect. All measures examining negative outcomes loaded significantly onto the latent ill-being factor.

## Discussion

The present study aimed to extend on limited research into need frustration within the educational and workplace setting. We also sought to further justify the separation between need frustration and satisfaction put forward by Longo et al. (2016). Further, due to the prevalence of mental illness within the educational (American College Health Association, 2018) and workplace setting (Bonde, 2008; Fan et al., 2015), we examined the relationship between psychological health problems and need frustration.

In support of hypothesis one, positive correlations between the measures assessing ill-being and frustration occurred. Further, the negative correlation with these measures and satisfaction increases support for this hypothesis. Measures of negative affect, depression, stress and most measures of anxiety, were also moderately positively correlated with relatedness and competence frustration. These measures were similarly negatively associated with relatedness and competence satisfaction. In most cases, ill-being measures were only weakly correlated with autonomy satisfaction and frustration. This suggests that psychological health problems might not be strongly related to feelings of autonomy. With regard to the educational setting, this is plausible, considering that autonomy has been found to be a weak predictor of positive outcomes, such as academic motivation within undergraduate populations (Grolnick et al., 2002; Faye and Sharpe, 2008).

Hypothesis two was also supported as a positive relationship was found between positive affect and satisfaction, whereas this relationship was negative with frustration. Like the measures of ill-being, autonomy satisfaction had the weakest relationship with positive affect. Future research should seek to extend the examination of autonomy beyond the undergraduate population, through testing post-graduate students. Within the workplace, a need for autonomy is a critical predictor of well-being, performance, motivation, and reduced emotional distress (Gagné and Bhave, 2011). Post-graduate students are expected to feel a stronger need for autonomy than undergraduate students, through increased control over output, therefore the satisfaction and frustration of autonomy might be more important to this population.

Hypothesis three was partially supported. Relatedness satisfaction and competence satisfaction significantly loaded onto positive affect, and relatedness frustration and competence frustration loaded significantly onto ill-being. However, autonomy satisfaction did not significantly load onto positive affect, and autonomy frustration did not load significantly onto ill-being.

In the original study conducted by Longo et al. (2016), additional measures of vigor, intrinsic motivation and job satisfaction, were examined in relation to their relationship with wellbeing. This study did not include these measures and therefore, only examined the relationship between positive affect and need satisfaction. This could have reduced potential factor loadings between autonomy satisfaction and positive affect, as autonomy satisfaction might be more strongly related to these additional measures. Indeed, the factor loading for autonomy satisfaction and well-being in the study by Longo et al. (2016) was higher and significant when these other measures were included. A similar proposition can explain the non-significant loading between autonomy frustration and ill-being. Despite this study extensively examining the relationship between ill-being outcomes, we did not include a measure assessing exhaustion. Therefore, autonomy frustration might be strongly related to exhaustion. Measures of exhaustion, intrinsic motivation, vigor and job satisfaction, should be included when examining the relationship with well-being and ill-being during future studies.

The finding that state somatic anxiety as measured by the STICSA significantly loaded onto ill-being and that this was related to need frustration, is important theoretically. It suggests that need frustration is related to physiological anxiety symptoms such as automatic nervous system arousal (Ree et al., 2008). This supports Bartholomew et al. (2011b) who found that need thwarting (frustration) was related to somatic complaints in athletes. This is in extension of Longo et al.’s (2016) findings as they did not examine the relationship between the NSFS and somatic anxiety. It also elaborates on Bartholomew et al. as somatisation was found to influence need frustration outside the domain of athletes. Further, the significant relationship between ill-being and stress as measured by the DASS-Stress scale is also theoretically important, as this finding is novel, and therefore in extension of previous research (Longo et al., 2016).

Relatedness frustration had the strongest relationship with ill-being. This supports previous research (Larson et al., 1996). Negative affect and depression strongly, and significantly, loaded onto ill-being. Relatedness frustration directly relates to experiencing social exclusion and loneliness (Chen et al., 2015), with prolonged periods of loneliness associated with depression and increased negative affect (van Winkel et al., 2017). Interventions within the university setting should focus on instilling a greater sense of inclusion within university students, which in turn, might reduce symptoms of depression and negative affect. In the study conducted by Mattanah et al. (2010), students that partook in a peer-led social inclusion program, experienced increased feelings of social inclusion and reduced loneliness.

In addition to interventions specific to improving peer relationships, research has also highlighted to importance of the teacher in terms of fostering a sense of inclusion, with this subsequently, strengthening relatedness. Students reported increased relatedness when they felt that their teacher genuinely cared, respected and valued them (Niemiec and Ryan, 2009). Therefore, lecturers and tutors should endeavor to convey increased warmth, caring and respect toward students (Niemiec and Ryan, 2009). This finding also has implications for the workplace, as it has been found that transformational leaders, who foster relatedness, through increased employee respect, and through instilling a sense of cohesion through shared team goals, improved outcomes (Kovjanic et al., 2013). Therefore, transformational leadership training programs (Hasson et al., 2016) focusing on improving leader/followers’ relationships should be implemented (Dvir et al., 2002).

State cognitive anxiety as measured by the STICSA, was also strongly related to ill-being. After relatedness frustration, competence frustration was the strongest predictor of ill-being. Competence frustration relates to negative feelings an individual has toward their self-efficacy and increased feelings of failure (Sweet et al., 2012; Chen et al., 2015). Like with depression, increased anxiety is associated with low self-efficacy (Jerusalem and Schwarzer, 1992). Therefore, in addition to relatedness frustration increasing the manifestation of psychological health problems, competence frustration might also contribute to ill-being. Therefore, interventions within both the educational and workplace setting, should also target competence. According to Niemiec and Ryan (2009) competence within the educational setting can be improved through rewarding effort, in addition to academic performance. Some students report that despite immense effort, they do not receive the academic performance they expect, and therefore feel their effort has been under-rewarded (Copeland and Levesque-Bristol, 2011). This feeling of inadequacy consequently reduces competence. Presently, most scholarships within the university context are awarded based on academic merit. Despite grades reflecting motivation to learn in some cases, for some students, effort is more indicative of performance. Therefore, to instill a feeling of competence, some scholarships could be awarded to students based on the level of effort or degree of improvement a student makes (Copeland and Levesque-Bristol, 2011).

This study lends support to the proposition that need satisfaction and need frustration are separate constructs (Longo et al., 2016, 2018). Further, the finding that need frustration is strongly related to psychological health problems, specifically negative affect, depression, anxiety and stress, extends research into need frustration (Longo et al., 2016). Unlike Longo et al. (2016), this study separately measured the manifestation of state anxiety and depression symptoms created through increased need frustration. This study also examined the influence of need frustration on the expression of stress. The current study highlights the magnitude of potential ill-being outcomes created through increased frustration of psychological needs, specifically competence and relatedness frustration, expressed within the workplace and/or educational settings.

### Limitations and Future Directions

Potential limitations of this study are the lack of measures examining vigor and intrinsic motivation. Despite this study’s main aim seeking to extensively examine the predictors of ill-being, future research should include more well-being measures. This will allow for a deeper examination into the claim that need frustration and need satisfaction are distinct constructs instead of need frustration relating to the absence of need satisfaction (Longo et al., 2016, 2018). Further, a measure of exhaustion should be included to examine whether autonomy frustration is a significant predictor of Need Frustration, or if its inclusion in the NSFS should be reviewed.

Future research should seek to implement the suggested interventions related to reducing competence and relatedness frustration within both the workplace and university setting. Within the educational setting, it was recommended that depression and negative affect could be reduced through peer-led social inclusion programs fostering social inclusion and reducing isolation (Mattanah et al., 2010). Rewarding effort, rather than academic merit, via the implementation of effort-based scholarships (Copeland and Levesque-Bristol, 2011) might increasing self-efficacy and competence, subsequently decreasing anxiety and depression. Lastly, within both the educational and workplace setting, lecturers, tutors and leaders, could more outwardly express respect and value toward their students and employees to improve relatedness. To quantifying the magnitude of improvement once interventions are implemented, a longitudinal design should be used. Within the university setting, psychological need satisfaction/frustration could be measured when students first start university, to act a baseline, and measured once again after the implementation of interventions. To quantify the retention of positive outcomes after implementation, additional measures should be taken for the duration of the student’s undergraduate degree.

## Conclusion

The current study gives preliminary support to Longo et al. (2016, 2018), who stated that need frustration and need satisfaction are distinct constructs. Theoretically, this study also gives further insight into the relationship between basic need frustration and common types of psychological health problems, such as anxiety specific to physiological symptoms, and stress. Whilst practically, potential interventions to reduce need frustration and reduce psychological symptoms of ill-being are presented.

## Data Availability

The datasets generated for this study are available on request to the corresponding author.

## Ethics Statement

This study was carried out in accordance with the recommendations of the National Statement on Ethical Conduct in Human Research, 2007, National Health and Medical Research Council Act 1992, with written informed consent from all subjects. All subjects gave written informed consent in accordance with the Declaration of Helsinki. The protocol was approved by the Human Research Ethics Committee at Murdoch University.

## Author Contributions

IT collected and analyzed the data, and prepared the draft and final manuscript. GC provided feedback on draft manuscripts to prepare it for publication.

## Conflict of Interest Statement

The authors declare that the research was conducted in the absence of any commercial or financial relationships that could be construed as a potential conflict of interest.

## References

[B1] American College Health Association (2018). *American College Health Association-National College Health Assessment II: Undergraduate Student Reference Group Executive Summary Spring 2018.* Hanover: American College Health Association, 2018.

[B2] Baard P. P. Deci E. L. Ryan R. M. (2004). Intrinsic need satisfaction: a motivational basis of performance and weil ∼being in two work settings. *J. Appl. Soc. Psychol.* 34 2045–2068. 10.1111/j.1559-1816.2004.tb02690.x

[B3] Bartholomew K. J. Ntoumanis N. Ryan R. M. Bosch J. A. Thøgersen-Ntoumani C. (2011a). Self-determination theory and diminished functioning: the role of interpersonal control and psychological need thwarting. *Personal. Soc. Psychol. Bull.* 37 1459–1473. 10.1177/0146167211413125 21700794

[B4] Bartholomew K. Ntoumanis N. Ryan R. M. Thøgersen-Ntoumani C. (2011b). Psychological need thwarting in the sport context: assessing the darker side of athletic experience. *J. Sport Exerc. Psychol.* 33 75–102. 10.1123/jsep.33.1.75 21451172

[B5] Beck A. T. Steer R. A. Brown G. K. (1996). *Manual for the Beck Depression Inventory-*, Vol. II San Antonio, TX: Psychological Corporation, 78.

[B6] Bonde J. P. E. (2008). Psychosocial factors at work and risk of depression: a systematic review of the epidemiological evidence. *Occup. Environ. Med.* 65 438–445. 10.1136/oem.2007.038430 18417557

[B7] Chen B. Vansteenkiste M. Beyers W. Boone L. Deci E. L. Van der Kaap-Deeder J. (2015). Basic psychological need satisfaction, need frustration, and need strength across four cultures. *Motiv. Emot.* 39 216–236. 10.1007/s11031-014-9450-1

[B8] Church A. T. Katigbak M. S. Ibáñez-Reyes J. de Jesús Vargas-Flores J. Curtis G. J. Tanaka-Matsumi J. (2014). Relating self-concept consistency to hedonic and eudaimonic well-being in eight cultures. *J. Cross Cult. Psychol.* 45 695–712. 10.1177/0022022114527347

[B9] Clark L. A. Watson D. (1991). Tripartite model of anxiety and depression: psychometric evidence and taxonomic implications. *J. Abnorm. Psychol.* 100 316–336. 10.1037/0021-843X.100.3.316 1918611

[B10] Cole D. A. Ciesla J. A. Steiger J. H. (2007). The insidious effects of failing to include design-driven correlated residuals in latent-variable covariance structure analysis. *Psychol. Methods* 12 381–398. 10.1037/1082-989X.12.4.381 18179350

[B11] Colligan T. W. Higgins E. M. (2006). Workplace stress: etiology and consequences. *J. Workplace Behav. Health* 21 89–97. 10.1300/J490v21n02_07

[B12] Copeland K. J. Levesque-Bristol C. (2011). The retention dilemma: effectively reaching the first-year university student. *J. Coll. Stud. Ret.* 12 485–515. 10.2190/CS.12.4.f

[B13] Cronbach L. J. (1951). Coefficient alpha and the internal structure of tests. *Psychometrika* 16 297–334. 10.1007/bf02310555

[B14] Danhauer S. C. Legault C. Bandos H. Kidwell K. Costantino J. Vaughan L. (2013). Positive and negative affect, depression, and cognitive processes in the cognition in the Study of tamoxifen and raloxifene (Co-STAR) Trial. *Aging Neuropsychol. Cogn.* 20 532–552. 10.1080/13825585.2012.747671 23237718 PMC3815441

[B15] Deci E. L. Ryan R. M. (2000). The “what” and “why” of goal pursuits: human needs and the self-determination of behavior. *Psychol. Inquiry* 11 227–268. 10.1207/S15327965PLI1104_01 20204932

[B16] Deci E, L. Ryan R. M. (eds). (2002). *Handbook of Self-Determination Research*. Rochester, NY: University of Rochester Press.

[B17] Dozois D. J. Dobson K. S. Ahnberg J. L. (1998). A psychometric evaluation of the beck depression inventory–II. *Psychol. Assess.* 10 83–89. 10.1037/1040-3590.10.2.83

[B18] Dvir T. Eden D. Avolio B. J. Shamir B. (2002). Impact of transformational leadership on follower development and performance: a field experiment. *Acad. Manag. J.* 45 735–744. 10.5465/3069307

[B19] Fan L. B. Blumenthal J. A. Watkins L. L. Sherwood A. (2015). Work and home stress: associations with anxiety and depression symptoms. *Occup. Med.* 65 110–116. 10.1093/occmed/kqu181 25589707 PMC4402380

[B20] Faye C. Sharpe D. (2008). Academic motivation in university: the role of basic psychological needs and identity formation. *Can. J. Behav. Sci.* 40 189–199. 10.1037/a0012858

[B21] Field A. (2009). *Discovering Statistics Using SPSS*, 3rd Edn London: Sage Publications Ltd.

[B22] Gagné M. Bhave D. (2011). *“Autonomy in the Workplace: An Essential Ingredient to Employee Engagement and Well-being in Every Culture”. In Human autonomy in cross-cultural context.* Dordrecht: Springer, 163–187.

[B23] Gagné M. Chemolli E. Forest J. Koestner R. (2008). A temporal analysis of the relation between organisational commitment and work motivation. *Psychol. Belg.* 48 219–241. 10.5334/pb-48-2-3-219

[B24] Gagné M. Senécal C. B. Koestner R. (1997). Proximal job characteristics. Feelings of empowerment, and intrinsic motivation: a multidimensional model. *J. Appl. Soc. Psychol.* 21 1222–1240. 10.1111/j.1559-1816.1997.tb01803.x

[B25] Ghasemi A. Zahediasl S. (2012). Normality tests for statistical analysis: a guide for non-statisticians. *Int. J. Endocrinol. Metab.* 10 486–489. 10.5812/ijem.3505 23843808 PMC3693611

[B26] Goldberg D. Williams P. D. P. M. (1988). *A User’s Guide to the General Health Questionnaire. 1988.* Windsor: NFER-Nelson.

[B27] Grolnick W. S. Gurland S. T. DeCourcey W. Jacob K. (2002). Antecedents and consequences of mothers’ autonomy support: an experimental investigation. *Dev. Psychol.* 38 143–155. 10.1037/0012-1649.38.1.143 11806696

[B28] Grös D. F. Antony M. M. Simms L. J. McCabe R. E. (2007). Psychometric properties of the state-trait inventory for cognitive and somatic anxiety (sticsa): comparison to the state-trait anxiety inventory (STAI). *Psychol. Assess.* 19 369–381. 10.1037/1040-3590.19.4.369 18085930

[B29] Hair J. F. Anderson R. E. Tatham R. L. Black W. C. (1995). *Multivariate Data Analysis.* Englewood Cliffs, NJ: Prentice-Hall.

[B30] Hasson H. von Thiele Schwarz U. Holmstrom S. Karanika-Murray M. Tafvelin S. (2016). Improving organizational learning through leadership training. *J. Workplace Learn.* 28 115–129. 10.1108/JWL-06-2015-0049

[B31] Hooper D. Coughlan J. Mullen M. (2008). Structural equation modelling: guidelines for determining model fit. *Electron. J. Buis. Res. Methids* 6 53–60. 10.1016/j.acap.2015.07.001 26547545

[B32] Hu L. Bentler P. M. (1999). Cutoff criteria for fit indexes in covariance structure analysis: conventional criteria versus new alternatives, structural equation modeling: a Multidisciplinary. *Journal* 6 1–55. 10.1080/10705519909540118

[B33] Jerusalem M. Schwarzer R. (1992). “Self-efficacy as a resource factor in stress appraisal processes”, in *Self-efficacy: Thought Control of Action*, ed. Schwarzer R. (Washington DC: Hemisphere), 195–213.

[B34] Kline R. B. (1998). *Principle and Practice of Structural Equation Modeling*. New York, NY: Guilford Publications.

[B35] Kovjanic S. Schuh S. C. Jonas K. (2013). Transformational leadership and performance: an experimental investigation of the mediating effects of basic needs satisfaction and work engagement. *J. Occup. Organ. Psychol.* 86 543–555. 10.1111/joop.12022

[B36] Larson R. W. Richards M. H. Moneta G. Holmbeck G. Duckett E. (1996). Changes in adolescents’ daily interactions with their families from ages 10 to 18: disengagement and transformation. *Dev. Psychol.* 32 744–754. 10.1037/0012-1649.32.4.744

[B37] Little R. J. A. (1988). A test of missing completely at random for multivariate data with missing values. *J. Am. Statist. Assoct.* 83 1198–1202. 10.1080/01621459.1988.10478722

[B38] Longley S. L. Watson D. Noyes R. Jr. (2005). Assessment of hypochondriasis domain: the multidimensional inventory of hypochondriacal traits (MIHT). *Psychol. Assess.* 17 3–14. 10.1037/1040-3590.17.1.3 15769224

[B39] Longo Y. Alcaraz-Ibáñez M. Sicilia A. (2018). Evidence supporting need satisfaction and frustration as two distinguishable constructs. *Psicothema* 30 74–81. 10.7334/psicothema2016.367 29363474

[B40] Longo Y. Gunz A. Curtis G. J. Farsides T. (2016). Measuring need satisfaction and frustration in educational and work contexts: the need satisfaction and frustration scale (NSFS). *J. Happiness. Stud.* 17 295–317. 10.1007/s10902-014-9595-3

[B41] Lovibond P. F. Lovibond S. H. (1995). The structure of negative emotional states: comparison of the depression anxiety stress scales (DASS) with the beck depression and anxiety inventories. *Behav. Res. Ther.* 33 335–343. 10.1016/0005-7967(94)00075-U 7726811

[B42] Mattanah J. F. Ayers J. F. Brand B. L. Brooks L. J. Quimby J. L. McNary S. W. (2010). A social support intervention to ease the college transition: exploring main effects and moderators. *J. Coll. Stud. Deve.* 51 93–108. 10.1353/csd.0.0116

[B43] Niemiec C. P. Ryan R. M. (2009). Autonomy, competence, and relatedness in the classroom: applying self-determination theory to educational practice. *School Field* 7 133–144. 10.1177/1477878509104318

[B44] Peterson R. A. Heilbronner R. L. (1987). The anxiety sensitivity index:: construct validity and factor analytic structure. *J. Anxiety Disord.* 1 117–121. 10.1016/0887-6185(87)90002-8

[B45] Ree M. J. French D. MacLeod C. Locke V. (2008). Distinguishing cognitive and somatic dimensions of state and trait anxiety: development and validation of the state-trait inventory for cognitive and somatic anxiety (STICSA). *Behav. Cogn. Psychother.* 36 313–332. 10.1017/S1352465808004232

[B46] Reiss S. Peterson R. A. Gursky D. M. McNally R. J. (1986). Anxiety sensitivity, anxiety frequency and the prediction of fearfulness. *Behav. Res. Ther.* 24 1–8. 10.1016/0005-7967(86)90143-9 3947307

[B47] Riolli L. Savicki V. Richards J. (2012). Psychological capital as a buffer to student stress. *Psychology* 3 1202–1207. 10.4236/psych.2012.312A178

[B48] Roberts K. E. Hart T. A. Eastwood J. D. (2016). Factor structure and validity of the state-trait inventory for cognitive and somatic anxiety. *Psychol. Assess.* 28 134–146. 10.1037/pas0000155 26011481

[B49] Ryan R. M. Sheldon K. M. Kasser T. Deci E. L. (1996). “All goals are not created equal: an organismic perspective on the nature of goals and their regulation,” in *The psychology of action: Liking cognitivion to Behaviour*, eds Gollwitzer P. M. Bargh J. A. (New York, N.Y: Guildford Press), 7–26.

[B50] Sheldon K. M. Gunz A. (2009). Psychological needs as basic motives, not just experiential requirements. *J. Pers.* 77 1467–1492. 10.1111/j.1467-6494.2009.00589.x 19678877

[B51] Spielberger C. D. Gorsuch R. L. Lushene R. Vagg P. R. Jacobs G. A. (1983). *Manual for the State-Trait Anxiety Inventory.* Palo Alto, CA: Consulting Psychologists Press.

[B52] Spielberger C. D. Gorsuch R. L. Lushene R. E. (1970). *STAI: Manual for the STATE-Trait Anxiety Inventory.* Palo Alto, CA: Consulting Psychologists Press.

[B53] Sweet S. N. Fortier M. S. Strachan S. M. Blanchard C. M. (2012). Testing and integrating self-determination theory and self-efficacy theory in a physical activity context. *Can. Psychol. Psychol. Can.* 53 319–327. 10.1037/a0030280

[B54] van Winkel M. Wichers M. Collip D. Jacobs N. Derom C. Thiery E. (2017). Unraveling the role of loneliness in depression: the relationship between daily life experience and behavior. *Psychiatry* 80 104–117. 10.1080/00332747.2016.1256143 28767331

[B55] Vansteenkiste M. Ryan R. M. (2013). On psychological growth and vulnerability: basic psychological need satisfaction and need frustration as a unifying principle. *J. Psychother. Int.* 23 263–280. 10.1037/a0032359

[B56] Watson D. Clark L. A. (1994). *The PANAS-X: Manual for the Positive and Negative Affect Schedule-Expanded Form.* Ames: The University of Iowa.

